# Development and Validation of a Hypoxia-Associated Prognostic Signature Related to Osteosarcoma Metastasis and Immune Infiltration

**DOI:** 10.3389/fcell.2021.633607

**Published:** 2021-03-18

**Authors:** Yucheng Fu, Qiyuan Bao, Zhuochao Liu, Guoyu He, Junxiang Wen, Qi Liu, Yiqi Xu, Zhijian Jin, Weibin Zhang

**Affiliations:** Department of Orthopedics, Ruijin Hospital, School of Medicine, Shanghai Jiao Tong University, Shanghai, China

**Keywords:** osteosarcoma, hypoxia, prognosis, metastasis, immune

## Abstract

**Background:**

Increasing evidence has shown that hypoxia microenvironment relates to tumor initiation and progression. However, no studies focus on the application of hypoxia-associated genes in predicting osteosarcoma patients’ prognosis. This research aims to identify the hypoxia-associated genes related to osteosarcoma metastasis and construct a gene signature to predict osteosarcoma prognosis.

**Methods:**

The differentially expressed messenger RNAs (DEmRNAs) related to osteosarcoma metastasis were identified from Therapeutically Applicable Research to Generate Effective Treatments (Target) database. Univariate and multivariate cox regression analyses were performed to develop the hypoxia-associated prognostic signature. The Kaplan–Meier (KM) survival analyses of patients with high and low hypoxia risk scores were conducted. The nomogram was constructed and the gene signature was validated in the external Gene Expression Omnibus (GEO) cohort. Single-sample gene set enrichment analysis (ssGSEA) was conducted to investigate the relationships between immune infiltration and gene signature.

**Results:**

Two genes, including decorin (DCN) and prolyl 4-hydroxylase subunit alpha 1 (P4HA1), were involved in the hypoxia-associated gene signature. In training and testing datasets, patients with high-risk scores showed lower survival rates and the gene signature was identified as the independent prognostic factor. Receiver operating characteristic (ROC) curves demonstrated the robustness of signature. Functional analyses of DEmRNAs among high- and low-risk groups revealed that immune-associated functions and pathways were significantly enriched. Furthermore, ssGSEA showed that five immune cells (DCs, macrophages, neutrophils, pDCs, and TIL) and three immune features (CCR, APC co inhibition, and Check-point) were down-regulated in the high-risk group.

**Conclusion:**

The current study established and validated a novel hypoxia-associated gene signature in osteosarcoma. It could act as a prognostic biomarker and serve as therapeutic guidance in clinical applications.

## Introduction

Osteosarcoma is one of the most common primary bone malignant tumors which predominately occurs in the juvenile population (Ritter and Bielack, 2010). With the progression of multimodal treatment, especially neoadjuvant chemotherapy combined with wide surgical excision, the 5-year survival rates of these patients have significantly improved to over 70% (Anderson, 2016). However, a large number of patients present with metastasis at initial diagnosis or after intensive treatment. Less than one-fifth of these patients could survive over 5 years (Dai et al., 2011; Kansara et al., 2014). Therefore, targeting osteosarcoma metastasis has been a hot direction and numerous researchers focus on it. Unfortunately, little progress has been achieved since the underlying mechanisms are still unclear.

Hypoxia, or oxygen deficiency, is one of the hallmarks of human solid tumor (Ruan et al., 2009). It relates to rapid proliferation of tumor cells which triggers the imbalance between oxygen demand and supply. During malignant tumor progression, hypoxia always interacts with other hallmarks and enhances epithelial-mesenchymal transition (EMT), angiogenesis, and stemness of tumors (Mathieu et al., 2011; Guo et al., 2020; Hapke and Haake, 2020). Recent studies also indicate that hypoxia could influence tumor immune microenvironment, such as decreasing natural killer (NK) cell and cytotoxic T lymphocyte (CTL) activity, increasing immunosuppressive cells (i.e., TAMs, Tregs, and MDSCs) differentiation potential, and enhancing disadvantageous immune cytokines expression (Terry et al., 2017). All these biological processes finally lead to resistance to chemotherapy, distal metastasis, and poor prognosis of patients (Vaupel, 2008; Walsh et al., 2014).

With the development of microarray and next-generation sequencing technology, numerous aberrantly expressed oncogenes are detected. The majority of these genes relate to occurrence or development of cancers and some could serve as the prognostic signature (Liu et al., 2020; Wu et al., 2020). In osteosarcoma, several gene signatures related to energy metabolism, tumor microenvironment and immune system have been investigated (Hong et al., 2020; Hu et al., 2020; Zhu et al., 2020). However, no hypoxia-associated prognostic signature has been established.

**FIGURE 1:**
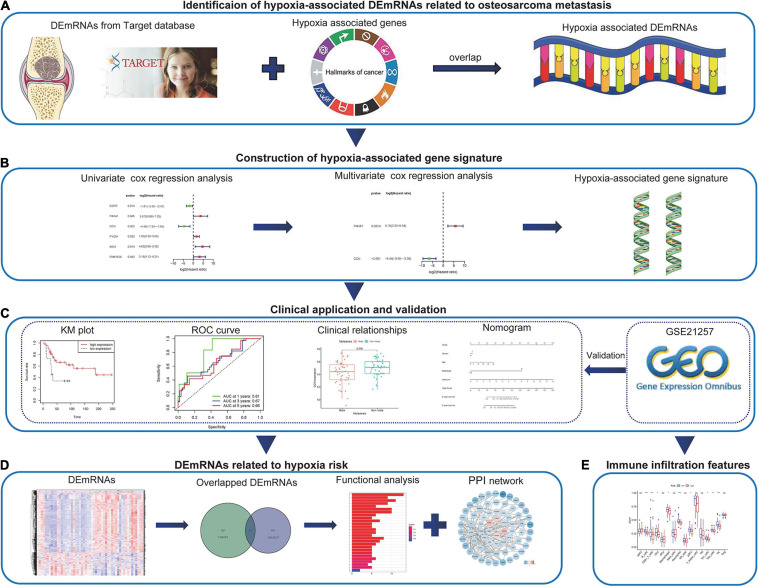
The work-flow of the study. **(A)** The hypoxia-associated metastatic DEmRNAs were identified through the Target and MSigDB databases. **(B)** Combined methods were used to construct the hypoxia-associated gene signature. **(C)** The application of the hypoxia-associated gene signature in clinic and validation in GEO database. **(D)** Identification and functional analysis of DEmRNAs associated with hypoxia risk. **(E)** Immune infiltration features associated with hypoxia risk. DEmRNA, differentially expressed messenger RNA; GEO, Gene Expression Omnibus.

In the present study, we obtained hypoxia-associated differentially expressed messenger RNAs (DEmRNA) among metastatic and non-metastatic patients in Therapeutically Applicable Research to Generate Effective Treatments (Target) database. DEmRNAs related to prognosis were screened out through univariate cox regression analysis and the prognostic signature was obtained by multivariate cox regression analysis. Then the Kaplan–Meier (KM) survival analyses of patients with high and low signature risk scores were conducted. The nomogram was built and the signature’s relationship with immune infiltration was also explored. What is more, the prognostic value of the signature was validated in the Gene Expression Omnibus (GEO) database. The work-flow of this study is shown in Figure 1. We hoped that the present research would extend our knowledge between osteosarcoma metastasis and hypoxia. We believed that the gene signature could serve as a promising prognostic biomarker and might act as a potential therapeutic target for osteosarcoma patients.

## Materials and Methods

### Selection of Datasets

The mRNA expression profiles of osteosarcoma were searched from GEO^1^ and Target^2^ databases. The datasets involved in this study should meet the following criteria: (1) the tumors were confirmed as osteosarcoma by histology; (2) the datasets contained metastatic and non-metastatic patients; (3) the datasets had complete prognosis-associated information; (4) the sample size in the dataset was more than 50. At last, the osteosarcoma RNA-seq from Target was selected as the training group and GSE21257 (platform GPL10295, Illumina human-6 v2.0 expression beadchip Illumina, Inc., San Diego, CA, United States) was used as the validation group. The clinical characteristics of patients in Target and GSE21257 datasets are shown in Supplementary Table 1. The hypoxia-associated genes were downloaded from the hallmark gene sets in the Molecular Signature Database^3^ (MSigDB).

### Acquisition of Hypoxia-Associated DEmRNAs

Patients’ mRNA expression data were downloaded from target database and merged as the file through Perl software. The clinical information was also obtained from Target database and then patients were divided into metastatic and non-metastatic groups based on their features. The Linear Models for Microarray Analysis (limma) package was applied to perform the analysis of DEmRNAs between two groups. The cutoff value of DEmRNAs was *P* < 0.05 and | fold change (FC)| > 1.5. The volcano plot was drawn through the “pheatmap” package in R software. The hypoxia-associated DEmRNAs were obtained through the overlap between DEmRNAs and hypoxia-associated genes.

### Construction and Validation of the Hypoxia-Associated Prognostic Signature

The hypoxia-associated prognostic genes were selected from univariate cox regression analysis by overall survival (OS). The multivariate cox regression analysis was conducted to identify the independent prognostic genes and the prognostic signature was established based on the multivariate cox stepwise regression analysis. The minimum number of mRNAs that represented the signature was screened through Akaike information criterion (AIC) (Vrieze, 2012). Then the risk score was calculated through multivariate Cox regression model coefficients multiplied by gene expression values as follows:

$$\mathrm{Risk}\,\mathrm{score}=\sum_{i}{\text{Coefficient}}_{\text{mRNAi}}*{\mathrm{Expression}}_{\text{mRNAi}}$$

All patients with complete prognostic data were divided into high- and low-risk groups based on the median value of risk score. The KM survival analysis of hypoxia-associated signature in the whole and subgroups were conducted through the “survival” package in R software. The time-related receiver operating characteristic (ROC) curves were performed to test the predictive value of this gene signature.

Next, univariate and multivariate cox regressions were used again to verify the independent risk role of this hypoxia-associated gene signature in clinical application. The subgroup analyses of individual genes in the hypoxia-associated prognostic signature were conducted based on patients’ clinical features. The hypoxia-associated signature was validated in GSE21257. All above processes were operated in R software. *P* < 0.05 was considered as statistically significant.

### Construction of the Nomogram

In order to predict the 3- and 5-year survival probability of osteosarcoma patients, the nomogram was established based on risk scores and clinical factors such as age, gender, and metastasis (Iasonos et al., 2008). Each factor in the nomogram was assigned a score based on the results of the multivariate cox regression analysis. Harrell’s concordance index (C-index) was conducted to estimate the prediction bias of the nomogram. The nomogram was validated through GSE21257 once more. The package “rms” in R software was used to operate above tasks.

### Identification and Functional Enrichment Analyses of Gene Signature-Related DEmRNAs

In the Target and GSE21257 datasets, DEmRNAs between high- and low-risk groups were obtained through the “limma” package in R software. The heatmap was drawn by the “pheatmap” package. The overlapping DEmRNAs in two datasets were identified as the risk score-associated DEmRNAs.

To study the interactions between DEmRNAs, the online tool Search Tool for the Retrieval of Interacting Genes database^4^ (STRING) was used to construct the protein–protein interaction (PPI) network (Szklarczyk et al., 2015). Then the network was visualized in Cytoscape (v3.7.1) and the disconnected nodes were hidden. Gene ontology (GO) functional and Kyoto Encyclopedia of Genes and Genomes (KEGG) pathway enrichment analyses of risk score-associated DEmRNAs were performed by “clusterProfiler” and “enrichplot” packages in R software. *P*- and *q*-values of <0.05 were defined as significantly enriched.

### Estimation of Immune Infiltration Status Related to Hypoxia

The immune infiltration of 16 immune cells and 13 immune-associated features in the Target and GSE21257 datasets were evaluated through single-sample gene set enrichment analysis (ssGSEA). The overlapped items occurring with the same trends were considered as the immune characteristic changes. The R package “gsva” was used and *P* < 0.05 was considered as statistically significant.

## Results

### Identification of Hypoxia-Associated DEmRNAs Related to Osteosarcoma Metastasis

**FIGURE 2:**
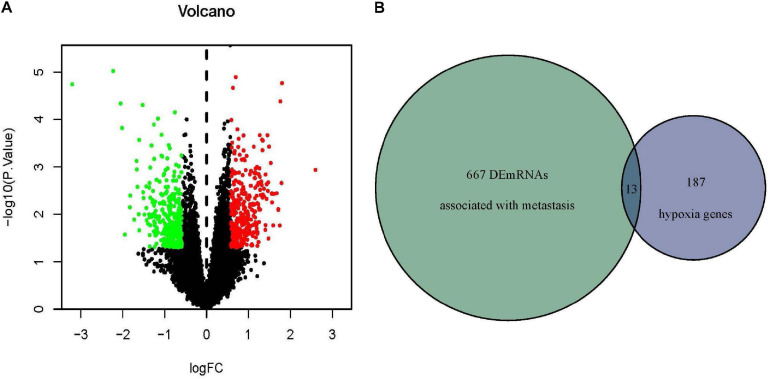
Identification of hypoxia-associated DEmRNAs related to osteosarcoma metastasis. **(A)** DEmRNAs related to osteosarcoma metastasis in Target database. Red indicated upregulated DEmRNAs (*P* < 0.05, FC > 1.5) and green indicated downregulated DEmRNAs (P > 0.05, FC < –1.5). **(B)** Thirteen overlapped hypoxia-associated DEmRNAs were identified in the Target and MSigDB databases. DEmRNA, differentially expressed messenger RNA; FC, fold change.

The mRNA expression data were downloaded from the Target dataset which contained 54 metastatic and 43 non-metastatic osteosarcoma patients. 680 DEmRNAs (329 up-regulated and 351 down-regulated) associated with metastasis were identified and the volcano plot showed the distribution trend (Figure 2A). The hypoxia-related genes were obtained from MSigDB and then overlapped with DEmRNAs obtained previously (Figure 2B). At last, 13 hypoxia-associated DEmRNAs were screened out (Table 1).

**TABLE 1:** The hypoxia-associated DEmRNAs in Target dataset.

Gene	Log_2_FC	*P* value
DCN	−0.65	0.031
EFNA1	0.74	0.00016
EGFR	−0.98	0.0036
ENO2	0.67	0.021
FAM162A	0.65	0.0021
MXI1	0.70	0.000013
NCAN	−0.70	0.040
NDRG1	0.69	0.0031
P4HA1	0.70	0.00071
PGF	0.81	0.0022
PYGM	1.21	0.0058
SCARB1	0.66	0.0023
TPD52	0.89	0.0057

*DEmRNA, differentially expressed messenger RNA.*

### Construction of the Hypoxia-Associated Prognostic Signature in Target Database

**FIGURE 3:**
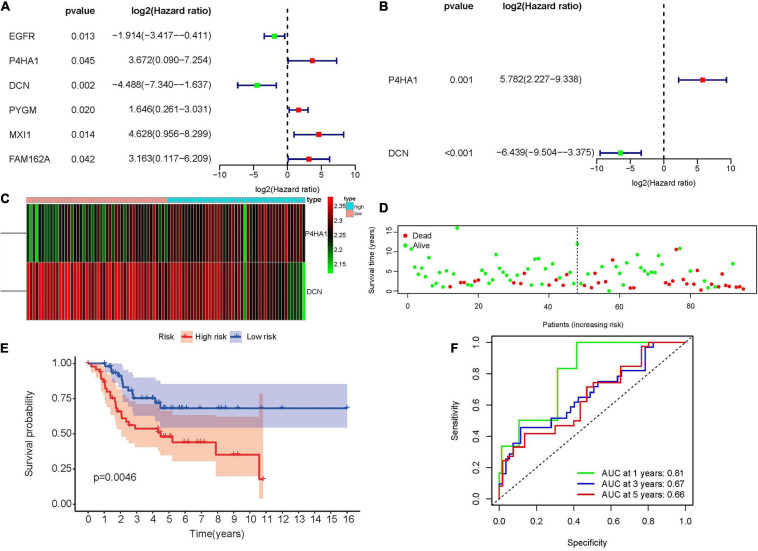
Construction of the hypoxia-associated prognostic signature in the Target database. **(A)** Univariate cox regression analysis of hypoxia-associated DEmRNAs revealed six prognostic genes. **(B)** Multivariate cox regression analysis revealed two independent hypoxia-associated DEmRNAs. **(C)** The heatmap of P4HA1 and DCN in high- and low-risk groups showed that P4HA1 was positively correlated with risk score while DCN showed the opposite trend. **(D)** The scatter plot of patients’ risk scores and survival time. **(E)** Kaplan–Meier survival plot of patients in high- and low-risk groups. **(F)** Time-dependent ROC curve at 1, 3, and 5 years of hypoxia-associated prognostic signature. DEmiRNA, differentially expressed microRNA; ROC, receiver operating characteristic; P4HA1, prolyl 4-hydroxylase subunit alpha 1; DCN, decorin.

To construct the hypoxia-associated gene signature in osteosarcoma, univariate and multivariate cox regression analyses were performed in 13 hypoxia-related DEmRNAs. In univariate cox regression analysis, six genes showed significant relationships with osteosarcoma patients’ OS (Figure 3A). Base on the multivariate cox regression analysis, two genes were identified as the independent prognostic factors (Figure 3B) and the risk score formula was established just as mentioned previously:

Risk⁢score=(4.01*P4HA1)+(-4.46*DCN).

According to the median value of the risk scores, 47 patients were identified as high-risk and the rest, 48, were considered as low-risk (Supplementary Figure 1). The expression of prolyl 4-hydroxylase subunit alpha 1 (P4HA1) was positively correlated with risk score while decorin (DCN) showed the opposite trend (Figure 3C). Patients with low-risk scores appeared to have lower mortality rates and longer survival years than those with high-risk scores (Figure 3D). At the same time, the KM survival curve also indicated that the high-risk group had worse prognoses (Figure 3E, *p* = 0.0046). In the end, the predictive accuracy of the risk score was performed through time-dependent ROC curve. The areas under the ROC curve were 0.81 at 1 year, 0.67 at 3 year, and 0.66 at 5 year, which indicated that the risk model was reliable (Figure 3F).

### Validation of the Hypoxia-Associated Prognostic Signature in GSE21257

**FIGURE 4:**
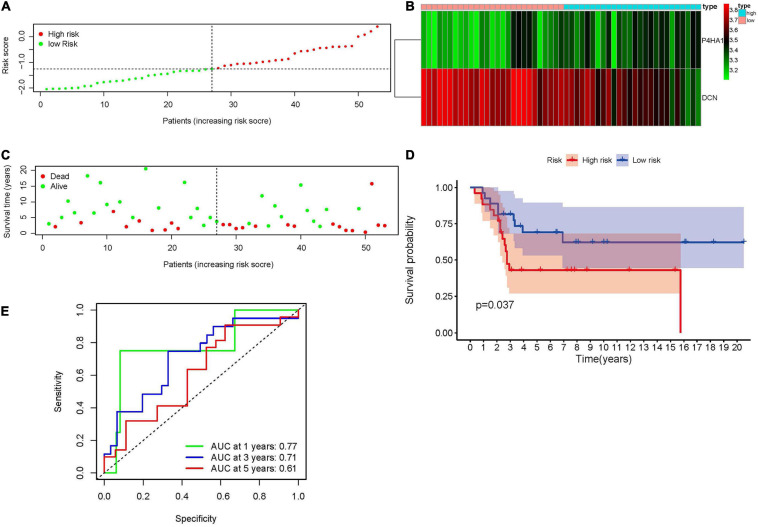
Validation of the hypoxia-associated prognostic signature in GSE21257. **(A)** The distribution and median value of risk score. **(B)** The expression of P4HA1 and DCN in high- and low-risk groups revealed that P4HA1 was positively correlated with risk score while DCN showed the opposite trend. **(C)** The scatter plot of patients associated with risk scores. **(D)** Kaplan–Meier survival plot of patients in high- and low-risk groups. **(E)** Time-dependent ROC curve at 1, 3, and 5 years of hypoxia-associated prognostic signature. ROC, receiver operating characteristic; P4HA1, prolyl 4-hydroxylase subunit alpha 1; DCN, decorin.

The robustness of the hypoxia-associated prognostic signature was tested in GSE21257. A total of 53 patients with integrated survival data were enrolled in this validation group. Patients were separated into high- and low-risk groups according to the median value of previous formula results (Figure 4A). The expression trends of prognostic genes in the GSE21257 dataset were similar to the Target database (Figure 4B). At the same time, the risk plot (Figure 4C) and KM survival analysis (Figure 4D, *p* = 0.037) also indicated that a high risk score was related to poor prognosis. Moreover, the time-dependent ROC curve demonstrated that the prognostic signature was convincible (Figure 4E).

### Correlations Between Hypoxia-Associated Gene Signature and Clinical Parameters

**FIGURE 5:**
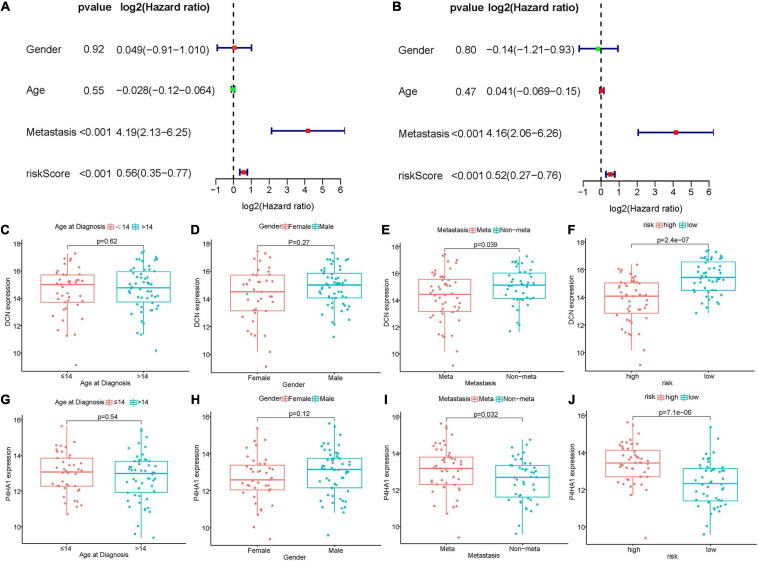
Relationships between hypoxia-associated gene signature and clinical parameters in Target dataset. **(A,B)** Univariate and multivariate cox regression analyses revealed risk score and metastasis were two independent prognostic factors. **(C–F)** Boxplot of DCN expression and clinical characteristics. **(G–J)** The correlation between P4HA1 expression and clinical characteristics. DCN, decorin; P4HA1, prolyl 4-hydroxylase subunit alpha 1.

To evaluate the prognostic value of hypoxia-associated gene signature in clinical application, univariate and multivariate cox regression analyses were performed once more. As shown in Figures 5A and B, risk score (HR = 1.43, 95% CI = 1.20–1.69, *P* < 0.001) was regarded as the independent prognostic factor in Target dataset. The same results were obtained in GSE21257 (Supplementary Figures 2A,B).

The KM survival analyses of gene signature in different clinical subgroups were also conducted. As shown in Supplementary Figures 3A–E, high risk scores related to worse survival rate in metastasis (*P* = 0.043), less or equal to 14 years (*P* = 0.040), over 14 years (*P* = 0.012), female (*P* = 0.017) and male (*P* = 0.036) groups. The same results were identified in GSE21257 (Supplementary Figures 4A–E).

The relationships between prognostic signature genes and clinical parameters were performed through Wilcoxon rank-sum test. In the Target matrix, the expression of P4HA1 and DCN were not related to the patient’s age at diagnosis or gender (Figures 5C,D,G,H). In contrast, P4HA1 significantly increased in metastatic (*p* = 0.032) and high-risk (*p* < 0.01) groups (Figures 5I,J), while DCN showed the opposite trend (and Figures 5E,F). The same results also appeared in the GSE21257 dataset except that the metastasis status was not correlated with two gene expression (Supplementary Figures 2C–J).

### Construction and Validation of the Nomogram

**FIGURE 6:**
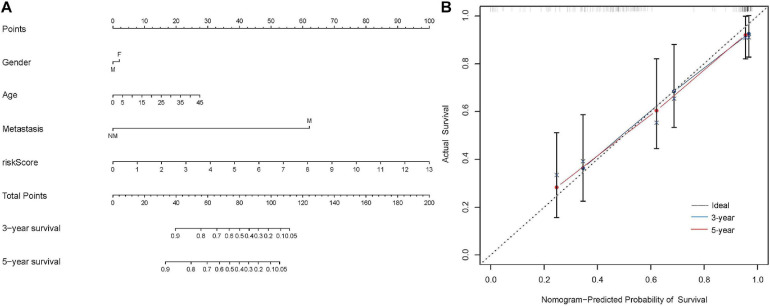
Construction of the nomogram in the Target dataset. **(A)** The nomogram to predict the 3- and 5-year survival risk of osteosarcoma patients. **(B)** The calibration curve of the 3- and 5-year survival in Target dataset.

Nomogram was a powerful tool that integrated different risk factors to predict the prognosis of patients. In the present study, the nomogram associated with osteosarcoma patients’ survival was established in the Target dataset (Figure 6A). The C-index (0.85) and calibration curve indicated the high reliability of the nomogram (Figure 6B). The same results were achieved in the validation matrix GSE21257 (Supplementary Figures 5A,B).

### Identification and Functional Analyses of DEmRNAs Related to Hypoxia-Associated Gene Signature

**FIGURE 7:**
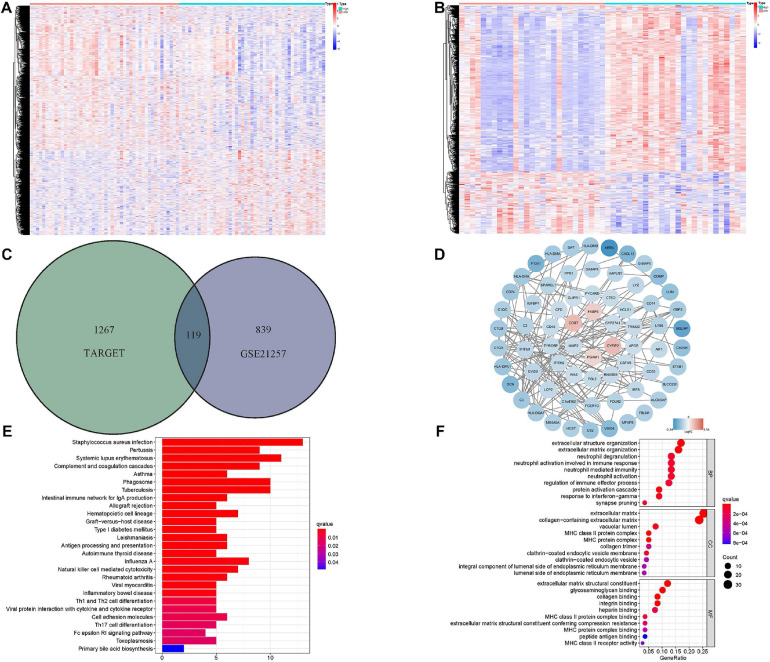
Identification and functional analyses of DEmRNAs related to hypoxia-associated gene signature. **(A)** The heatmap of risk score-associated DEmRNAs in Target dataset. **(B)** The heatmap of risk score-associated DEmRNAs in GSE21257. **(C)** The overlapped DEmRNAs between Target and GSE21257 datasets. **(D)** The PPI network of DEmRNAs. **(E,F)** KEGG and top 10 GO functional analyses results of DEmRNAs. DEmRNA: differentially expressed messenger RNA, PPI, protein–protein interaction; GO, gene ontology; KEGG, Kyoto encyclopedia of genes and genomes.

The DEmRNAs related to the hypoxia-associated prognostic signature were also investigated in the Target and GSE21257 datasets. About 1,386 mRNAs in the Target matrix and 958 mRNAs in GSE21257 were differentially expressed and the overlapped genes (119 DEmRNAs) were extracted for further analyses (Figures 7A–C). The interactions of DEmRNAs were established through the online tool STRING and the network was visualized in Cytoscape (Figure 7D).

In order to understand the potential biological functions of these DEmRNAs, GO functional and KEGG pathway analyses were conducted (Figures 7E,F). Numerous biological processes (i.e., extracellular matrix structural constituent, collagen binding, and extracellular structure organization) related to hypoxia were significantly enriched. It was worth noting that various immune-associated functions were also enriched, which indicated the widespread relevance between hypoxia and immune status.

### Evaluation of the Relationship Between Hypoxia and Immune Infiltration

**FIGURE 8:**
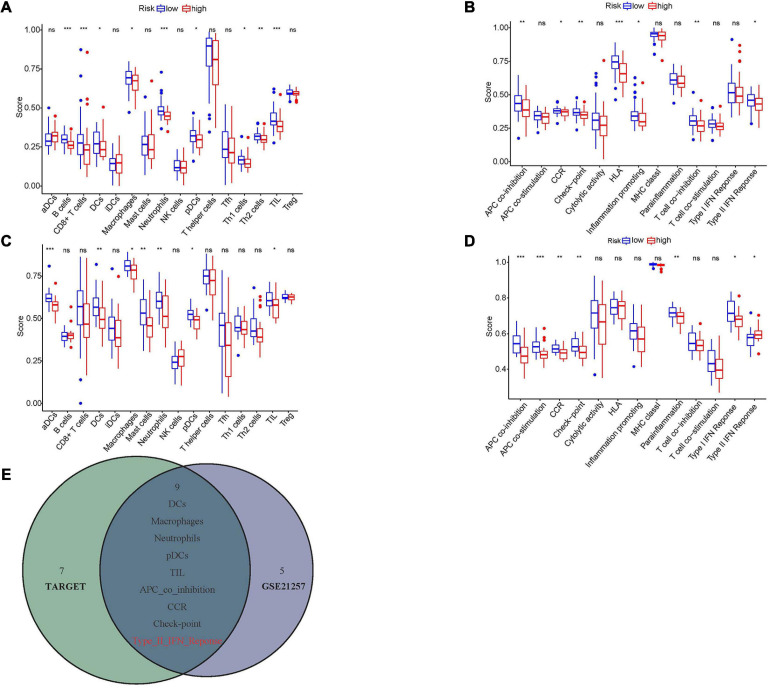
The relationships between hypoxia and immune infiltration. **(A,C)** The correlations between risk score and different immune cells in Target and GSE21257 datasets. **(B,D)** The association between risk score and different immune features in Target and GSE21257 datasets. **(E)** The overlapped immune cells and functions among Target and GSE21257 datasets. The red color implied the opposite trend in two datasets. NS, not significant; *P < 0.05; **P < 0.01; ***P < 0.001.

To further understand the association between hypoxia and immune infiltration, ssGSEA analysis was carried out. Nine immune cells and seven immune functions were significantly related to the hypoxia-associated risk score in the Target dataset (Figures 8A,B). At the same time, seven immune cells and seven immune functions were statistically significant in the GSE21257 dataset (Figures 8C,D). The intersection of two datasets contained five immune cells (DCs, macrophages, neutrophils, pDCs, and TIL) and four immune functions (CCR, APC co inhibition, Check-point, and Type II IFN response), while the Type II IFN response showed opposite trends in two databases and was eliminated (Figure 8E).

## Discussion

Osteosarcoma was one of the highly aggressive tumors which frequently developed metastasis. Lung was the most common metastatic site and the 5-year survival rates of these patients were extremely low (Isakoff et al., 2015). The mechanisms underlying tumor metastasis were first mentioned by Paget in 1889, just known as the “seed and soil” theory (Fokas et al., 2007). Since then, numerous hallmarks associated with tumor metastasis were proposed, such as gene fusion and mutation, EMT, angiogenesis and so on (Han et al., 2019). Among these risk factors, hypoxia was widely explored and considered as the crucial prognostic factor.

The contradiction between growing demands and inadequate supplement of blood always resulted in a hypoxia tumor microenvironment (Petrova et al., 2018). Under the hypoxia microenvironment, the tumor often initiated multiple adaptive transformations (i.e., migration, proliferation, and invasion) which eventually contributed to progression (Muz et al., 2015; Jing et al., 2019). In the last few decades, a large number of genes related to hypoxia have been identified in various cancers including osteosarcoma (Guo et al., 2014; Leng et al., 2018). For example, Cao et al. (2015) found that the expression of WSB1 in osteosarcoma was elevated under a hypoxia microenvironment, which promoted RhoGDI2 degradation and enhanced metastasis. What is more, many researchers realized that these genes could unite as the hypoxia-associated signature, which might serve as the prognostic biomarker in cancers (Mo et al., 2020; Zhang et al., 2020). However, no such signature had been built in osteosarcoma.

In the present study, we identified 680 DEmRNAs among metastatic and non-metastatic osteosarcoma patients in the Target dataset. The hypoxia-associated genes were screened out and the prognostic signature was established through univariate and multivariate cox regression analyses. Then we divided the cohort into high- and low-risk groups according to the risk scores. The KM survival analyses showed that high-risk patients lived for a shorter amount of time and the ROC curve indicated that the prognostic signature is robust. These results were validated in the dataset GES21257. What is more, we demonstrated that the risk score was the independent prognostic factor in both datasets. The KM plot of patients in different subgroups also indicated that the low-risk patients had better prognosis, which demonstrated the extensive applicability of the gene signature. At last, the nomogram including several clinical features and risk scores was constructed to estimate the prognosis of patients. The C-index and calibration curve indicated that the nomogram was robust, which further supported the reliability of this hypoxia-associated prognostic signature. To sum up, we believed that the hypoxia gene signature in this research was a convincible prognosis biomarker and could be applied in clinic.

The immune system was one of the major components in the tumor microenvironment and was often suppressed in hypoxia (Wigerup et al., 2016). Previous studies indicated that hypoxia promoted osteosarcoma development through increasing immunomodulatory proteins such as macrophage migration inhibitory factor (MIF), Galectin-1, and so on (Pierrevelcin et al., 2020; Song et al., 2020). In the present study, we identified the overlapped DEmRNAs related to the hypoxia-associated prognostic signature in the Target and GSE21257 datasets. The functional analyses of these DEmRNAs showed that they were enriched in several immune-associated functions and pathways, such as MHC class II protein complex binding, antigen processing presentation, Th1 and Th2 cell differentiation, and NK cell mediated cytotoxicity. Interestingly, numerous researchers also revealed the same results. For instance, Yamada et al. (2012) reported that the relevance between osteosarcoma and NK cells declined when NKG2D ligand MICA was decreased. This change eventually minimized the cytotoxicity of NK cells (Yamada et al., 2012). To further detect the correlations between hypoxia and immune infiltration, ssGSEA analysis was conducted. The results showed that five immune cells (DCs, macrophages, neutrophils, pDCs, and TIL) infiltrated lower in high-risk group. Among them, DCs were known as the antigen-presenting cells that often stimulated the differentiation of naïve T cells to eliminate tumors. In osteosarcoma, the hypoxia-associated factor HIF-1α inhibited DC functions, which impeded antitumor immunity (den Haan et al., 2000; Vaupel and Multhoff, 2018). Furthermore, the differentiation process of plasmacytoid DC (pDC) was also abolished by HIF-1α, which resulted in tumor progression (Labiano et al., 2015). The tumor infiltration lymphocyte (TIL), always regarded as the antitumor cell, was also exhausted in the osteosarcoma microenvironment and accelerated tumor recurrence (Shi et al., 2020). These results highlighted that the hypoxia microenvironment might down-regulate anti-tumor immune cells, which enhanced the immune escape of osteosarcoma and promoted metastasis as well as progression.

What is more, two genes involved in our hypoxia-associated prognostic signature also played crucial roles in cancer development. DCN, one of the small leucine-rich proteoglycans, constituted the extracellular matrix (ECM) and played key roles in stromal structure regulation. Recent research has revealed that DCN could act as the ligand to bind several receptor tyrosine kinases (RTK) in cancers, which inhibited tumorigenesis, angiogenesis, and immunomodulatory function (Sainio and Järveläinen, 2019; Xiao et al., 2020). DCN was also able to elevate cyclin kinase inhibitor P21 and apoptosis factors. These changes would suppress tumor growth and promote apoptosis (De Luca et al., 1996; Seidler et al., 2006). In osteosarcoma, Shintani et al. (2008) reported that DCN interacted with fibronectin (FN) and inhibited pulmonary metastasis. P4HA1 was the most common subtype of prolyl 4-hydroxylase which enhanced collagen modification through increasing 4-hydroxyproline (Chen et al., 2006). Numerous studies revealed that P4HA1 might act as the oncogenic factor. For example, P4HA1 could regulate the secretion of collagen in fibroblast and altered the composition of ECM. These alterations influenced cancer cells’ motility, adhesion, and morphology (Gilkes et al., 2013). Under a hypoxia microenvironment, the elevated HIF-1α induced the expression of P4HA1, which remodeled ECM and promoted tumor invasion, EMT, angiogenesis, and so on (Gilkes et al., 2013; Balamurugan, 2016). Interestingly, P4HA1 could stabilize HIF-1α in turn and then increased downstream genes expression (Xiong et al., 2018).

As far as we know, this was the first study that revealed the hypoxia-associated gene signature in osteosarcoma metastasis. We thought the gene signature might serve as the prognostic biomarker in clinical application. However, some limitations should be noticed. First, the cohort size was relatively small which probably resulted from the low incidence of osteosarcoma and lack of studies. Second, the specific mechanisms of DCN and P4HA1 in osteosarcoma were still unclear. Most importantly, our research was a retrospective investigation and further prospective studies should be designed to validate the results.

## Conclusion

In summary, we constructed and validated a novel hypoxia-related prognostic signature associated with osteosarcoma metastasis. Besides, we found that hypoxia was closely related to immune infiltration of osteosarcoma. This hypoxia gene signature could serve as the prognostic biomarker and inspired new thoughts in hypoxia- or immune-targeted therapy. More studies should be carried out to verify our findings and clarify the fundamental mechanisms of hypoxia in osteosarcoma.

## Data Availability Statement

The original contributions presented in the study are included in the article/Supplementary Material, further inquiries can be directed to the corresponding author/s.

## Author Contributions

YF and WZ designed the study. QL and QB searched the data from database. YF, JW, and QB performed analysis of the data *in silico*. YF and ZL wrote the manuscript. YX and ZJ revised the manuscript. WZ modified the language. All authors had read and approved the final manuscript.

## Conflict of Interest

The authors declare that the research was conducted in the absence of any commercial or financial relationships that could be construed as a potential conflict of interest.

## Footnotes

1 http://www.ncbi.nlm.nih.gov/geo

2 https://ocg.cancer.gov/programs/target

3 https://www.gsea-msigdb.org/gsea/msigdb/

4 https://string-db.org/
