# Supplementary material for: Development and Validation of a Hypoxia-Associated Prognostic Signature Related to Osteosarcoma Metastasis and Immune Infiltration
Source: Front Cell Dev Biol. 2021 Mar 18;9:633607. doi: 10.3389/fcell.2021.633607 (PMC8012854; doi:10.3389/fcell.2021.633607)
Supplement: Supplementary file 1 [file Table_1.DOC]

**Supplementary Table 1.** **The clinical characteristics of patients in Target and GSE21257 datasets.**

| **Variable** | **Target** | **GSE21257** |
| --- | --- | --- |
| **Gender** |  |  |
| Male | 57 | 34 |
| Female | 40 | 19 |
| **Primary site** |  |  |
| Upper limbs | 7 | 8* |
| Lower limbs | 85 | 44 |
| Trunk | 5 | NA |
| **Metastasis status** |  |  |
| Non-metastasis | 43 | 19 |
| Metastasis | 54 | 34 |
| **Age at diagnosis (mean years)** |  |  |
| Non-metastasis | 16.76 | 19.44 |
| Metastasis | 14.41 | 18.30 |
| **Survival status** |  |  |
| Death | 38* | 23 |
| Alive | 58 | 30 |
| **Survival time** |  |  |
| ≥5 years | 29 | 24 |
| <5 years | 67* | 29 |

*Some patients’ data were not applicable. NA:not applicable.
