# Supplementary material for: Development and Validation of a Hypoxia-Associated Prognostic Signature Related to Osteosarcoma Metastasis and Immune Infiltration
Source: Front Cell Dev Biol. 2021 Mar 18;9:633607. doi: 10.3389/fcell.2021.633607 (PMC8012854; doi:10.3389/fcell.2021.633607)
Supplement: Supplementary file 2 [file Data_Sheet_1.pdf]

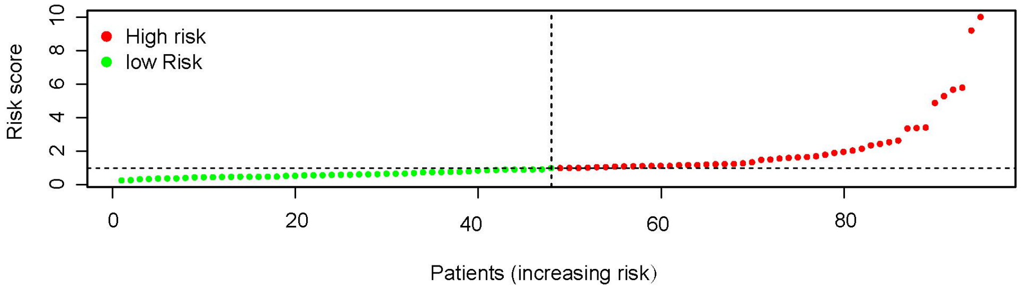

Supplementary Figure 1. The distribution and median value of risk score in Target database.

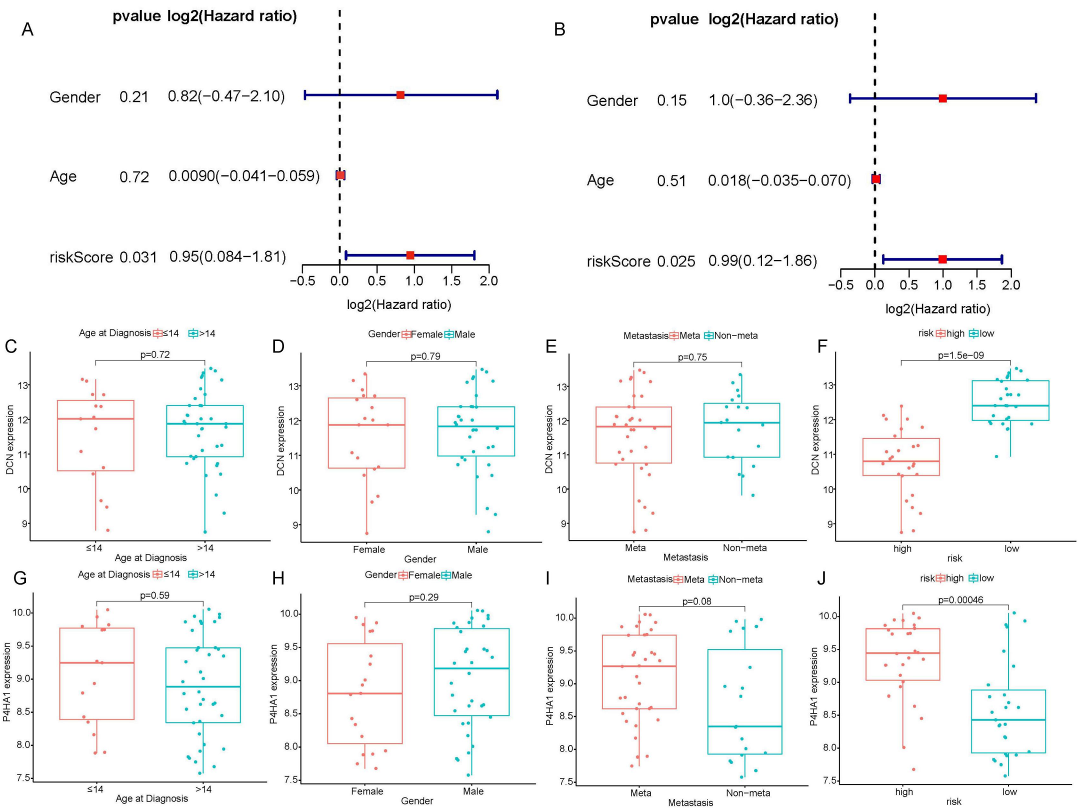

Supplementary Figure 2.Relationships between hypoxia-associated gene signature and clinical parameters in GSE21257. A and B.Univariate and multivariate cox regression analyses of risk score and clinical parameters. C-F. The association between DCN expression and clinical characteristics. G-J. The boxplot of P4HA1 expression and clinical characteristics. DCN: decorin, P4HA1: Prolyl 4-hydroxylase subunit alpha 1.

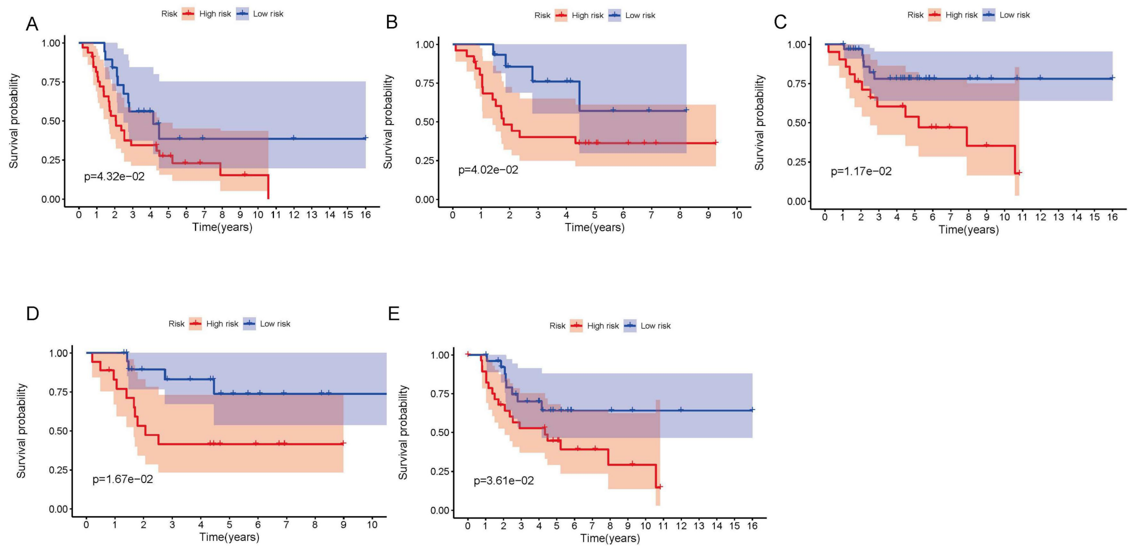

Supplementary Figure 3. The Kaplan-Meier survival plot of patients in metastasis(A), less or equal to 14 years(B), over 14 years(C), female(D) and male(D) subgroups in Target database.

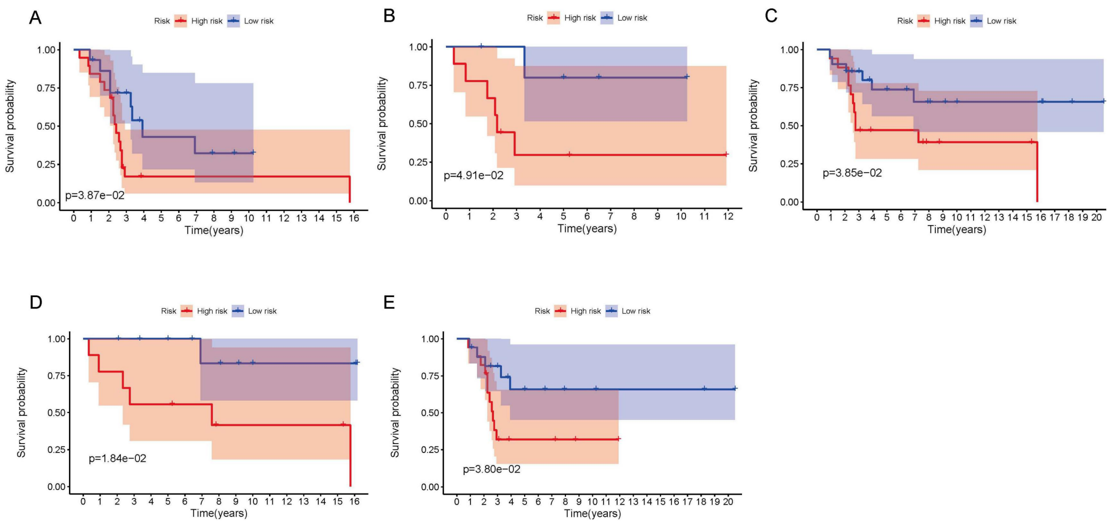

Supplementary Figure 4. The Kaplan-Meier survival plot of patients in metastasis(A), less or equal to 14 years(B), over 14 years(C), female(D) and male(D) subgroups in GSE21257.

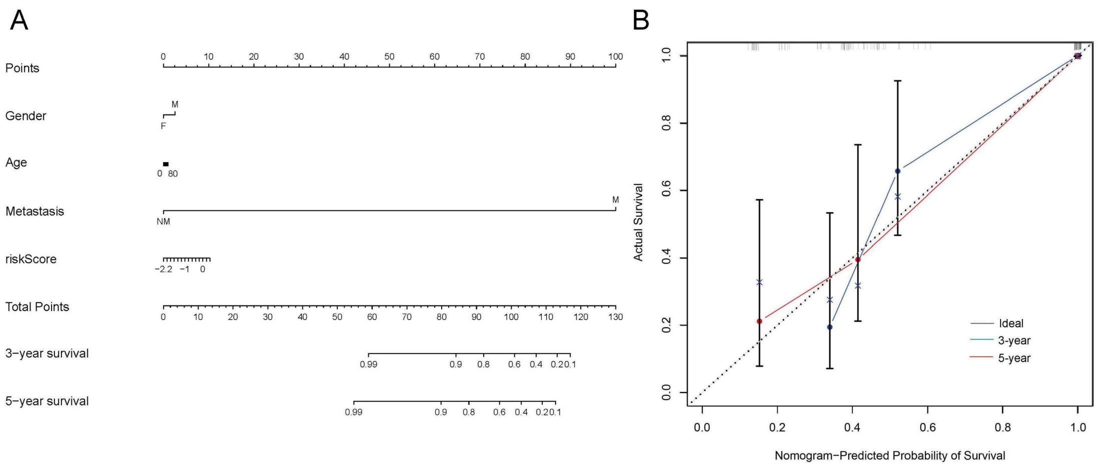

Supplementary Figure 5. Validation of the nomogram in GSE21257. A.The nomogram to predict the 3- and 5-year survival risk of osteosarcoma patients. B. The calibration curve of 3- and 5-year survival in GSE21257.
